# Mutations Associated with SARS-CoV-2 Variants of Concern, Benin, Early 2021

**DOI:** 10.3201/eid2711.211353

**Published:** 2021-11

**Authors:** Anna-Lena Sander, Anges Yadouleton, Edmilson F. de Oliveira Filho, Carine Tchibozo, Gildas Hounkanrin, Yvette Badou, Praise Adewumi, Keke K. René, Dossou Ange, Salifou Sourakatou, Eclou Sedjro, Melchior A. J. Aïssi, Hinson Fidelia, Mamoudou Harouna Djingarey, Michael Nagel, Wendy Karen Jo, Andres Moreira-Soto, Christian Drosten, Olfert Landt, Victor Max Corman, Benjamin Hounkpatin, Jan Felix Drexler

**Affiliations:** Charité-Universitätsmedizin Berlin, corporate member of Freie Universität Berlin, Humboldt-Universität zu Berlin, Institute of Virology, Berlin, Germany (A.-L. Sander, E.F. de Oliveira Filho, W.K. Jo, A. Moreira-Soto, C. Drosten, V.M. Corman, J.F. Drexler);; Ecole Normale Supérieure de Natitingou, Natitingou, Benin (A. Yadouleton);; Université Nationale des Sciences, Technologies, Ingénierie et Mathématiques (UNSTIM), Cotonou, Benin (A. Yadouleton);; Laboratoire des Fièvres Hémorragiques Virales du Benin, Cotonou (A. Yadouleton, C. Tchibozo, G. Hounkanrin, Y. Badou, P. Adewumi);; Ministry of Health, Cotonou (K.K. René, D. Ange, S. Sourakatou, B. Hounkpatin);; Conseil National de Lutte contre le VIH-Sida, la Tuberculose, le Paludisme, les IST et les Epidémies, Cotonou (E. Sedjro, M.A. Joël Aïssi, H. Fidelia);; World Health Organization Regional Office for Africa, Health Emergencies Programme, Brazzaville, Democratic Republic of the Congo (M.H. Djingarey);; Deutsche Gesellschaft für Internationale Zusammenarbeit, Bonn, Germany (M. Nagel);; German Centre for Infection Research (DZIF), associated partner Charité-Universitätsmedizin Berlin, Berlin (C. Drosten, V.M. Corman, J.F. Drexler);; TIB Molbiol Syntheselabor GmbH, Berlin (O. Landt)

**Keywords:** COVID-19, coronavirus disease, SARS-CoV-2, severe acute respiratory syndrome coronavirus 2, viruses, respiratory infections, zoonoses, variant of concern, mutations, Benin, West Africa

## Abstract

Intense transmission of severe acute respiratory syndrome coronavirus 2 (SARS-CoV-2) in Africa might promote emergence of variants. We describe 10 SARS-CoV-2 lineages in Benin during early 2021 that harbored mutations associated with variants of concern. Benin-derived SARS-CoV-2 strains were more efficiently neutralized by antibodies derived from vaccinees than patients, warranting accelerated vaccination in Africa.

Genomic surveillance is key to elucidate coronavirus disease (COVID-19) transmission chains and to monitor emerging severe acute respiratory syndrome coronavirus 2 (SARS-CoV-2) variants associated with partial or complete immune escape (*1*). Intense transmission likely promotes the emergence of variants, including mutations in the gene encoding the spike (S) protein, which is a major component of all available COVID-19 vaccines (*2*). Genomic surveillance is notoriously weak in sub-Saharan Africa (Appendix). A total of 55 SARS-CoV-2 lineages were described in West Africa as of May 25, 2021, considerably fewer than the >350 lineages in affluent regions (Appendix Figure, panel B). We previously described 2 diverse lineages (A.4 and B.1) in Benin early in the pandemic (*3*). In this study, we analyzed SARS-CoV-2 genomic diversity in Benin ≈1 year later and assessed the ability of vaccinee-derived and patient-derived serum samples to neutralize SARS-CoV-2 variants.

## The Study

We used 378 SARS-CoV-2–positive diagnostic respiratory samples tested at the reference laboratory in Benin during January 30–April 2, 2021, for genomic surveillance. All samples with cycle threshold <36 (Sarbeco E-gene assay; TIB Molbiol, https://www.tib-molbiol.de) were used for this study. To enable rapid prescreening of mutations known to affect the viral phenotype, we used 4 reverse transcription PCR (RT-PCR)–based single-nucleotide polymorphism (SNP) assays (VirSNiP; TIB Molbiol) targeting 9 hallmark mutations in 7 S codons of variants of concern (VOCs): B.1.1.7 (Alpha), B.1.351 (Beta), P.1 (Gamma), and B.1.617.2 (Delta) (Table 1). A total of 374 (98.9%) samples selected for the study tested positive for >1 mutation. Of those, ≈67.5% (255/378) showed the 69/70 deletion, 58.9% (223/378) the E484K mutation, 33.9% (128/378) the N501Y mutation, 30.4% (115/378) the P681H mutation, 14.8% (56/378) the L452R mutation, and 0.3% (1/378) the K417N or P681R mutation. The K417T or V1176F mutations associated with the Beta and Gamma VOCs were not detected. Approximately 22.2% (84/378) of samples were typeable to 1 of the lineages covered by the VirSNiP assays. According to SNP-based analyses, 14.8% (56/378) of the overall samples showed the mutation pattern of the Alpha variant, B.1.1.7, and 7.4% (28/378) of the B.1.525 variant. Frequent occurrence of the mutations under study suggests that earlier SARS-CoV-2 lineages not carrying those mutations have been replaced in Benin.

**Table 1:** Screened mutations, potential effects, and occurrence in severe acute respiratory syndrome coronavirus 2 variants, Benin, 2021

SNP assay	Spike protein variation	Potential effects	SARS-CoV-2 variant						
B.1.1.7 Alpha†	B.1.525	B.1.351 Beta†	P.1 Gamma†	P.2	P.3	B.1.617.2 Delta†
1	del HV69/70	Immune escape and enhanced viral infectivity (*4*)	x	x					
	E484K	Antibody resistance (*4*)		x	x	x	x	x	
	N501Y	Increased transmission (*4*)	x		x	x		x	
2	V1176F	Higher mortality rates‡				x	x		
3	L452R	Antibody resistance (*4*)							x
4	K417T	No data				x			
	K417N	Immune escape (*5*)			x				
	P681H	No data	x						
	P681R	No data							x

*SARS-CoV-2, severe acute respiratory syndrome coronavirus 2; SNP single-nucleotide polymorphism. †Variants of concern according to the World Health Organization. ‡G. Hahn et al., unpub. data, https://www.biorxiv.org/content/10.1101/2020.11.17.386714v2.

**Figure 1:**
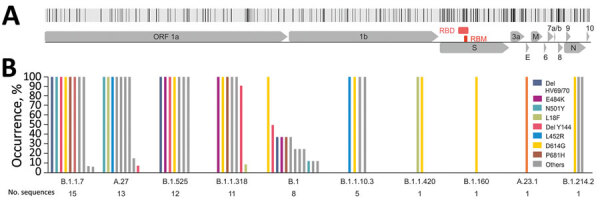
Genomic surveillance of severe acute respiratory syndrome coronavirus 2 (SARS-CoV-2) lineages in Benin, 2021. A) Nonsynonymous mutations of Benin-derived SARS-CoV-2 sequences across the full genome. B) Spike mutations occurring in the SARS-CoV-2 lineages circulating in Benin. Hallmark mutations of variants of concern are shown in color. Other mutations occurring in the Benin-derived sequences are depicted in gray and summarized as others. ORF, open reading frame; RBD, receptor-binding domain.

Definite lineage designation relies on the full genome sequence. We selected 68 (9 typeable and 59 nontypeable) samples according to unique mutational patterns covering the complete period of the study for a NimaGen/Illumina-based whole-genome sequencing workflow (Appendix). All near-full genomes generated within this study were deposited into GISAID (https://www.gisaid.org; accession nos. EPI_ISL_2932532–84 and EPI_ISL_2958658–72). Lineage assignment using the Pangolin COVID-19 Lineage Assigner version 3.0.2 (https://pangolin.cog-uk.io) confirmed SNP-based lineage prediction in all 9 typeable samples selected for whole-genome sequencing (Appendix). Despite robust lineage prediction based on unambiguous SNP-based results, our data demonstrate the limited use of VirSNiP assays for strain designation; however, these assays can detect relevant mutations of currently circulating variants. The 68 Benin-derived near-complete genomes were designated to 10 unique lineages, suggesting higher genetic diversity in Benin than ≈1 year before (*3*). During early 2021, lineages B.1.1.7 (22%), A.27 (19.1%), B.1.525 (17.6%), and B.1.1.318 (16.2%) were most prominent in Benin (Appendix). Despite presence of the mutation P681R (associated with the Delta VOC) in 1 sequence, that strain was typed as A.23.1, and no Delta variant was found. These data are consistent with recent online sequence reports from West Africa (A.E. Augustin, unpub. data, https://www.medrxiv.org/content/10.1101/2021.05.06.21256282v1; E.A. Ozer et al., unpub. data, https://www.medrxiv.org/content/10.1101/2021.04.09.21255206v3). A 100% consensus sequence of all 68 Benin-derived sequences showed 229 nonsynonymous nucleotide substitutions across the whole genome; 57 (24.9%) occurred in the S protein (Figure 1, panel A). Of note, variants with mutations in the S protein might alter the transmissibility and antigenicity of the virus (*4*). Internationally recognized VOCs to date share 16 S mutations in unique combinations (https://covariants.org/shared-mutations). The Benin-derived SARS-CoV-2 strains shared 10 unique S mutations reported in VOCs, although most of those strains were not defined as any VOC other than Alpha (Figure 1, panel B), suggesting convergent evolution of key mutations across different lineages (D.P. Martin et al., unpub. data, https://www.medrxiv.org/content/10.1101/2021.02.23.21252268v3; S. Cherian, unpub. data, https://www.biorxiv.org/content/10.1101/2021.04.22.440932v2). Putative higher fitness mediated by genomic change was consistent with more mutations in predominant lineages than in lineages found at lower frequencies (Figure 1, panel B).

**Figure 2:**
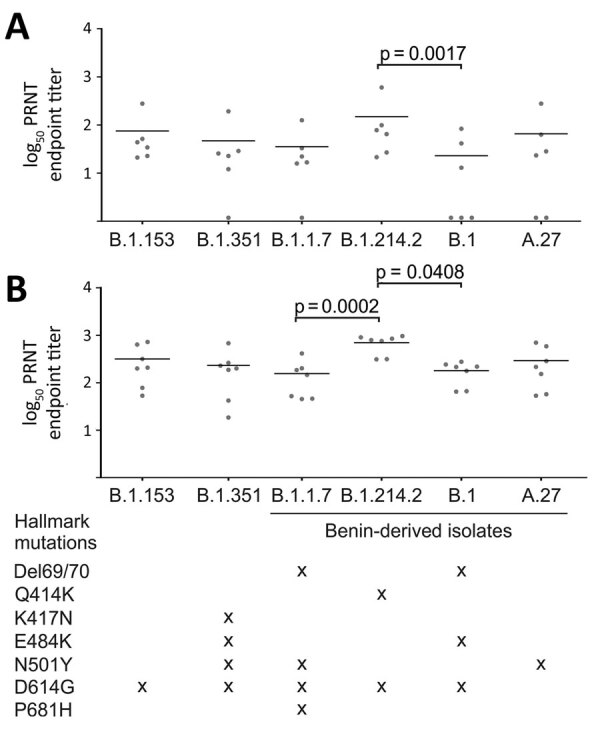
PRNT results of severe acute respiratory syndrome coronavirus 2 (SARS-CoV-2) variants from Benin, 2021. Graphs compare results of neutralization tests for naturally infected persons (A) and persons who received the Pfizer-BioNTech vaccine (BNT162b2; https://www.pfizer.com) (B) against the B.1.153 lineage from January 2020 (Munich/ChVir929/2020 strain; GISAID [http://www.gisaid.org] accession no. EPI_ISL_406862; Pangolin version 2021–05–19), the Beta stain (Baden-Wuertemberg/ChVir22131/2021; accession no. EPI_ISL_862149; B.1.351; Pangolin version 2021–05–19) and the B.1.1.7, B.1.214.2, B.1, and A.27 lineages isolated from patients from Benin. Lines denote the mean PRNT_50_ endpoint titer. Statistical significance was determined by the Dunn’s multiple comparisons test. Nonsignificant values are not shown for clarity of presentation. PRNT_50_, 50% plaque reduction neutralization test.

Because S mutations, individually or in combination, have been shown to afford viral escape to antibody-mediated immune responses, the high prevalence of variants with large numbers of these mutations circulating in Benin was cause for concern. To investigate whether and to what extent SARS-CoV-2 variants circulating in Benin and West Africa (*5*) evade neutralizing antibody responses, we isolated 4 lineages with unique mutational patterns (Table 2): an A.27 lineage isolate harboring the N501Y mutation; a B.1 isolate harboring the 69/70 deletion and the E484K and D614G mutations; a B.1.1.7 lineage isolate harboring the 69/70 deletion and the N501Y, D614G, and P681H mutations; and a B.1.214.2 lineage harboring the Q414K and D614G mutations (Figure 2). Additional isolation attempts of strains belonging to the frequently detected B.1.525 and B.1.318 lineages failed, likely because of degradation after repeated freeze-thaw cycles under tropical conditions. We tested neutralization potency of 6 serum samples from patients in Benin taken ≈8 days after RT-PCR–confirmed SARS-CoV-2 infection during early 2020 (*6*) and another 7 serum samples from persons in Europe 4 weeks after receiving the second dose of the Pfizer/BioNTech vaccine (BNT162b2; https://www.pfizer.com) (Appendix). Sampling was approved by the ethics committee of the Benin Ministry of Health (approval no. 030/MS/DC/SGM/DNSP/CJ/SA/027SGG2020) and of Charité-Universitätsmedizin Berlin (approval nos. EA1/068/20 and EA4/245/20). We compared neutralization titers with a SARS-CoV-2 strain (B.1.153) from January 2020 and the Beta strain (B.1.351), known to evade antibody-mediated neutralization (*7*). Despite the early sampling time after RT-PCR confirmation of SARS-CoV-2 infection, all 6 serum specimens from patients in Benin efficiently neutralized the early SARS-CoV-2 isolate carrying only the D614G mutation. In contrast, only 3 of those 6 serum specimens neutralized the B.1 isolate, the only isolate with the E484K mutation (Figure 2, panel A). Among the serum specimens from vaccinated persons, all neutralized the B.1 isolate, albeit at 1.5-fold lower titers than the early lineage B.1.153 isolate (by Friedman test and Dunn’s multiple comparisons test; p>0.99) (Figure 2, panel B). Those data were consistent with a recent report describing efficient neutralization of a B.1.525 strain from Nigeria by vaccinee-derived serum specimens (*8*). Of note, another strain classified as B.1.214.2 was neutralized more efficiently than all other tested lineages (Figure 2), highlighting that not every mutation in circulating lineages affords reduced antibody-mediated neutralization. Other hypothetically present fitness advantages of such strains will require detailed virologic investigation.

**Table 2:** Hallmark mutations and PRNT_50_ results of Benin-derived severe acute respiratory syndrome coronavirus 2 lineages, Benin, 2021

Sample no.	251307	314235	251455	312541
Lineage	B.1	B.1.1.7	A.27	B.1.214.2
Mutations	Q52R, Del HV69/70, Del Y144, E484K, D614G, Q677H, F888L	Del HV69/70, Del Y144, F490S, N501Y, A570D, D614G, P681H, T716I, S982A, D1118H	L18F, L452R, N501Y, A653V, H655Y, D796Y, G1219V	Ins R214TDR, Q414K, D614G, T716I
Patient-derived samples				
Mean titer (95% CI)	23 (–12.4 to 58.4)	35.5 (–12 to 83)	65.6 (–46.6 to 177.7)	148.9 (–86.59 to 384.3)
No. (%) neutralized	3/6 (50)	5/6 (83.3)	4/6 (66.7)	6/6 (100)
Titer difference†	52.2 (1.5-fold)	39.7	9.7	–73.6‡
Vaccinee-derived samples				
Mean titer (95% CI)	180.5 (102.8–258.1)	156.2 (33.6–278.7)	293.7 (57.1–530.2)	698.3 (446.8–949.9)
No. (%) neutralized	7/7 (100)	7/7 (100)	7/7 (100)	7/7 (100)
Titer difference†	136.7	161	23.5	–381.1‡

*PRNT_50_, 50% plaque reduction neutralization test. †Compared to variant B.1.153. ‡Lower titers against the early isolate compared with this Benin-derived isolate.

Our study is limited by patient-derived samples taken an average of 8 days after infection (*7*), which could imply incomplete maturation of antibodies. However, similar neutralization patterns between patient-derived and vaccinee-derived serum specimens suggest robustness of our data. Another limitation is that vaccinee-derived serum samples originated exclusively from Europe. Vaccine responses vary between populations, possibly influenced by genetic background and immune-modulating diseases (e.g., malaria or HIV) (*9*), highlighting the importance of testing serum samples from vaccinees in Africa for future studies. Of note, the efficacy trial of the Pfizer/BioNTech vaccine enrolled ≈40,000 participants, only ≈800 of whom were from Africa, and all of those from South Africa (*10*).

## Conclusions

Our data highlight the importance of ongoing monitoring of population immunity to emerging SARS-CoV-2 variants in Africa and of using serum specimens from local settings for phenotypic characterizations. Vaccination programs in Africa should be accelerated urgently, emphasizing the importance of global access to vaccines.

Appendix Additional information about mutations associated with SARS-CoV-2 variants of concern, Benin, early 2021
