# Supplementary material for: Mutations Associated with SARS-CoV-2 Variants of Concern, Benin, Early 2021
Source: Emerg Infect Dis. 2021 Nov;27(11):2889–903. doi: 10.3201/eid2711.211353 (PMC8544961; doi:10.3201/eid2711.211353)
Supplement: Appendix — Additional information about mutations associated with SARS-CoV-2 variants of concern, Benin, early 2021 [file 21-1353-Techapp-s1.pdf]

Article DOI: https://doi.org/10.3201/eid2711.211353

Mutations Associated with SARS-CoV-2 Variants of Concern, Benin, Early 2021

Appendix

Virus Isolation

Severe acute respiratory syndrome coronavirus 2 (SARS-CoV-2) isolation was performed by using 100µl of the reverse transcription PCR (RT-PCR)–positive nasal swab specimens diluted at 1:1 in OptiPro medium (ThermoFisher Scientific, https://www.thermofisher.com/us) and inoculated onto 1.5×10^5 Vero E6 cells, seeded in 24-well plates, maintained at 37°C, and cultivated in DMEM. After 1 h incubation, the inoculum was replaced by 500µl DMEM medium supplemented with 2% fetal calf serum (FCS), 1% nonessential amino acids, 1% Natrium Pyruvate, 1% Amphotericin B, 1% penicillin/Streptomycin (100 U/mL) (ThermoFisher Scientific). Infected cells were controlled daily for cytopathic effect (CPE) and passaged after 3 days. SARS-CoV-2 stocks were produced by propagation in Vero E6 cells. Cells and supernatant were harvested 3 days post-infection, centrifuged at 2500 × g for 10 min, and the supernatant titrated by plaque assay in Vero E6 cells.

Plaque Reduction Neutralization Test

Neutralizing antibodies against the different SARS-CoV-2 variants were detected by using a plaque reduction neutralization test ($PRNT_{50}$) conducted in monolayers of 1.6×10^5 Vero E6 cells, seeded in 12-well plates 1 day before the infection. Fifty plaque-forming units (PFUs) were incubated with serum dilutions of 1:20, 1:80, 1:320, and 1:1280 for 1 h, added onto the cell monolayer, and incubated again for 1 h before adding an overlayer containing DMEM with 1% FCS and 2% Avicell. After 2 days for B.1.153, B.1.351, and 3 days for the Benin isolates (B.1.1.7, B.1.214.2, B.1, and A.27), the overlayer medium was removed, and cells were fixated with 6% paraformaldehyde and stained with crystal violet. $PRNT_{50}$ endpoint titers were calculated by using a logistic regression function in GraphPad prism 6 (GraphPad Software. https://www.graphpad.com).

Appendix Table 1. Characteristics of severe acute respiratory syndrome coronavirus 2–positive samples from which full genomes were generated

ID	Collection date	C_t E-gene	Del HV69/70	E484K	N501Y	L452R	V1176F	K417N/T	P681H/R	Indicated variant	Pango lineage	Genome completeness, %
			Assay 1			Assay 2	Assay 3	Assay 4				
256146	2021 Feb 4	35.74	+	wt‡	+	wt‡	wt‡	wt‡	missing peak†	not typeable	B.1	91
315551	2021 Mar 3	17.88	+	wt‡	+	wt‡	wt‡	wt‡	+	B.1.1.7	B.1.1.7	99
314235	2021 Mar 2	15.76	+	wt‡	+	wt‡	wt‡	wt‡	+	B.1.1.7	B.1.1.7	100
255701	2021 Feb 4	17.85	+	wt‡	+	wt‡	wt‡	wt‡	+	B.1.1.7	B.1.1.7	100
255138	2021 Feb 3	26.62	+	wt‡	+	wt‡	wt‡	wt‡	+	B.1.1.7	B.1.1.7	99
253094	2021 Feb 2	18.84	+	wt‡	+	wt‡	wt‡	wt‡	+	B.1.1.7	B.1.1.7	100
251917	2021 Feb 1	20.57	+	wt‡	+	wt‡	wt‡	wt‡	+	B.1.1.7	B.1.1.7	100
251354	2021 Feb 1	19.88	+	wt‡	+	wt‡	wt‡	wt‡	+	B.1.1.7	B.1.1.7	100
251326	2021 Feb 1	15.97	+	wt‡	+	wt‡	wt‡	wt‡	+	B.1.1.7	B.1.1.7	100
311929	2021 Mar 2	23.94	+	wt‡	+	wt‡	wt‡	wt‡	missing peak†	not typeable	B.1.1.7	98
256208	2021 Feb 4	25.25	+	wt‡	+	wt‡	wt‡	wt‡	missing peak†	not typeable	B.1.1.7	100
256046	2021 Feb 4	24.86	+	wt‡	+	wt‡	wt‡	wt‡	missing peak†	not typeable	B.1.1.7	98
249234	2021 Jan 30	33.83	+	+	wt‡	wt‡	wt‡	wt‡	+	not typeable	B.1	97
251307	2021 Feb 1	17.21	+	+	wt‡	wt‡	wt‡	wt‡	missing peak†	not typeable	B.1	99
250990	2021 Feb 1	19.01	+	+	wt‡	wt‡	wt‡	wt‡	missing peak†	not typeable	B.1	96
312964	2021 Mar 2	31.63	+	+	wt‡	wt‡	wt‡	wt‡	missing peak†	not typeable	B.1.525	96
312950	2021 Mar 2	29.43	+	+	wt‡	wt‡	wt‡	wt‡	missing peak†	not typeable	B.1.525	98
312266	2021 Mar 1	17.89	+	+	wt‡	wt‡	wt‡	wt‡	missing peak†	not typeable	B.1.525	99
312198	2021 Mar 1	19.66	+	+	wt‡	wt‡	wt‡	wt‡	missing peak†	not typeable	B.1.525	99
254242	2021 Feb 3	17.73	+	+	wt‡	wt‡	wt‡	wt‡	missing peak†	not typeable	B.1.525	99
253408	2021 Feb 2	16.49	+	+	wt‡	wt‡	wt‡	wt‡	missing peak†	not typeable	B.1.525	99
250541	2021 Jan 31	21.35	+	+	wt‡	wt‡	wt‡	wt‡	missing peak†	not typeable	B.1.525	99
315465	2021 Mar 3	15.61	+	+	wt‡	wt‡	wt‡	wt‡	wt‡	B.1.525	B.1.525	99
254128	2021 Feb 3	18.45	wt‡	wt‡	wt‡	wt‡	wt‡	wt‡	681R	not typeable	A.23.1	99
253832	2021 Feb 2	24.05	wt‡	wt‡	wt‡	wt‡	wt‡	wt‡	+	not typeable	B.1	100
249868	2021 Jan 31	25.14	wt‡	wt‡	wt‡	wt‡	wt‡	wt‡	+	not typeable	B.1	100
249713	2021 Jan 31	25.52	wt‡	wt‡	wt‡	wt‡	wt‡	wt‡	+	not typeable	B.1	95
250814	2021 Feb 1	22.26	wt‡	wt‡	wt‡	wt‡	wt‡	wt‡	wt‡	no mutation	B.1	100
250323	2021 Jan 31	31.74	wt‡	wt‡	wt‡	wt‡	wt‡	wt‡	wt‡	no mutation	B.1.1.420	94
250412	2021 Feb 1	32.49	wt‡	wt‡	wt‡	wt‡	wt‡	wt‡	wt‡	no mutation	B.1.160	97
254278	2021 Feb 3	29.65	wt‡	wt‡	wt‡	+	wt‡	wt‡	wt‡	not typeable	L.3	99
250924	2021 Feb 1	21.61	wt‡	wt‡	wt‡	+	wt‡	wt‡	wt‡	not typeable	L.3	100
250772	2021 Feb 1	28.08	wt‡	wt‡	wt‡	+	wt‡	wt‡	wt‡	not typeable	L.3	99
249964	2021 Jan 31	31.88	wt‡	wt‡	wt‡	+	wt‡	wt‡	wt‡	not typeable	L.3	95
249110	2021 Jan 30	29.33	wt‡	wt‡	wt‡	+	wt‡	wt‡	wt‡	not typeable	L.3	98
250699	2021 Feb 1	22.88	wt‡	wt‡	+	+	n.e.	wt‡	wt‡	not typeable	A.27	100
312648	2021 Mar 1	28.07	wt‡	+	wt‡	wt‡	wt‡	n.e.	+	not typeable	B.1.1.318	97
312572	2021 Mar 1	21.97	+	wt‡	+	wt‡	wt‡	n.e.	+	not typeable	B.1.1.7	100
311995	2021 Mar 2	25.31	+	wt‡	+	wt‡	wt‡	neg	+	not typeable	B.1.1.7	99
314058	2021 Mar 2	21.58	n.e.	n.e.	+	wt‡	wt‡	wt‡	+	not typeable	B.1.1.7	100
312541	2021 Mar 1	23.00	wt‡	wt‡	wt‡	wt‡	wt‡	417N	wt‡	not typeable	B.1.214.2	98
248661	2021 Jan 30	32.81	+	+	wt‡	wt‡	n.e.	n.e.	missing peak†	not typeable	B.1.525	92
314176	2021 Mar 2	28.31	+	+	wt‡	wt‡	n.e.	n.e.	wt‡	not typeable	B.1.525	98
253620	2021 Feb 2	21.22	+	+	wt‡	wt‡	wt‡	wt‡	missing peak†	not typeable	B.1.525	98
254286	2021 Feb 3	23.32	+	+	wt‡	wt‡	n.e.	wt‡	missing peak†	not typeable	B.1.525	99
315530	2021 Mar 3	18.83	wt‡	+	wt‡	wt‡	wt‡	wt‡	+	not typeable	B.1.1.318	99

ID	Collection date	C_t E-gene	Del HV69/70	E484K	N501Y	L452R	V1176F	K417N/T	P681H/R	Indicated variant	Pango lineage	Genome completeness, %
			Assay 1			Assay 2	Assay 3	Assay 4				
312262	2021 Mar 1	18.44	wt‡	+	wt‡	wt‡	wt‡	wt‡	+	not typeable	B.1.1.318	99
312182	2021 Mar 1	16.95	wt‡	+	wt‡	wt‡	wt‡	wt‡	+	not typeable	B.1.1.318	99
311979	2021 Mar 2	18.25	wt‡	+	wt‡	wt‡	wt‡	wt‡	+	not typeable	B.1.1.318	94
254375	2021 Feb 3	20.15	wt‡	+	wt‡	wt‡	wt‡	wt‡	+	not typeable	B.1.1.318	99
253228	2021 Feb 2	21.04	wt‡	+	wt‡	wt‡	wt‡	wt‡	+	not typeable	B.1.1.318	99
249971	2021 Jan 31	20.47	wt‡	+	wt‡	wt‡	wt‡	wt‡	+	not typeable	B.1.1.318	99
249944	2021 Jan 31	22.11	wt‡	+	wt‡	wt‡	wt‡	wt‡	+	not typeable	B.1.1.318	99
248922	2021 Jan 30	21.60	wt‡	+	wt‡	wt‡	wt‡	wt‡	+	not typeable	B.1.1.318	99
251411	2021 Feb 1	24.71	wt‡	+	wt‡	wt‡	wt‡	wt‡	+	not typeable	B.1.1.318	96
255062	2021 Feb 3	17.53	wt‡	wt‡	+	+	wt‡	wt‡	missing peak†	not typeable	A.27	100
252348	2021 Feb 2	21.44	wt‡	wt‡	+	+	wt‡	wt‡	missing peak†	not typeable	A.27	98
312239	2021 Mar 1	24.61	wt‡	wt‡	+	+	wt‡	wt‡	wt‡	not typeable	A.27	100
255170	2021 Feb 3	25.66	wt‡	wt‡	+	+	wt‡	wt‡	wt‡	not typeable	A.27	100
254323	2021 Feb 3	19.68	wt‡	wt‡	+	+	wt‡	wt‡	wt‡	not typeable	A.27	100
253312	2021 Feb 2	19.71	wt‡	wt‡	+	+	wt‡	wt‡	wt‡	not typeable	A.27	100
251455	2021 Feb 1	19.76	wt‡	wt‡	+	+	wt‡	wt‡	wt‡	not typeable	A.27	99
251296	2021 Feb 1	20.07	wt‡	wt‡	+	+	wt‡	wt‡	wt‡	not typeable	A.27	100
250498	2021 Jan 31	20.67	wt‡	wt‡	+	+	wt‡	wt‡	wt‡	not typeable	A.27	100
250471	2021 Jan 31	31.86	wt‡	wt‡	+	+	wt‡	wt‡	wt‡	not typeable	A.27	98
249839	2021 Jan 31	22.66	wt‡	wt‡	+	+	wt‡	wt‡	wt‡	not typeable	A.27	100
248651	2021 Jan 30	23.22	wt‡	wt‡	+	+	wt‡	wt‡	wt‡	not typeable	A.27	98
314080	2021 Mar 2	21.90	wt‡	wt‡	+	wt‡	wt‡	wt‡	+	not typeable	B.1.1.7	100

*n.e., not evaluable; neg, negative; wt, wildtype.
†According to the manufacturer's instructions, missing peaks could be due to a drop out of the primer binding site
‡Sample does not harbor the tested mutation.

Appendix Table 2. Single-nucleotide polymorphism assay typing

Category	Typing based on SNP assay	No. samples typed	No. samples sequenced (%)	No. samples typing confirmed
Typeable	B.1.1.7	56	8 (14.3)	8
	B.1.525	28	1 (3.6)	1
	Total	84	9 (10.7)	9
Nontypeable	No mutation	4	3 (75)	n.a.
	Other variant†	337	56 (16.6)	n.a.
	Total	341	59	
Total		425	68	

*SNP, single-nucleotide polymorphism.

†Samples were categorized as other variant if any of the assays had negative or unclear results or if the mutational pattern did not enable typing to a specific lineage.

Appendix Table 3. Severe acute respiratory syndrome coronavirus 2 lineages to which the 68 Benin-derived full genomes were designated and the frequency of sequences within each lineage

Lineage	Number of sequences (%)
B.1.1.7	15 (22.0)
A.27	13 (19.1)
B.1.525	12 (17.6)
B.1.1.318	11 (16.2)
B.1	8 (11.8)
B.1.1.10.3	5 (7.4)
B.1.1.420	1 (1.5)
B.1.160	1 (1.5)
A.23.1	1 (1.5)
B.1.214.2	1 (1.5)

Appendix Table 4. Severe acute respiratory syndrome coronavirus 2 plaque reduction neutralization test titers ($PRNT_{50}$) of individual vaccinee- and patient-derived serum samples

Source	B.1.153	B.1.351	B.1.1.7	B.1.214.2	A.27	B.1
Patients						
Benin 1	34.3	22.8	33.0	78.1	23.4	13.0
Benin 2	278.1	192.0	125.4	602.8	278.5	83.4
Benin 3	43.7	12.1	16.7	64.8	28.4	neg
Benin 4	51.6	25.6	22.2	99.1	63.1	41.5
Benin 5	21.1	28.9	neg	21.5	neg	neg
Benin 6	22.8	neg	15.9	26.9	neg	neg
Mean	75.3	46.9	35.5	148.9	65.6	23.0
Vaccinees						
13684	200.4	198.0	185.3	790.5	588.2	215.2
13685	637.3	185.1	415.1	962.5	286.9	243.3
13686	53.4	18.1	52.1	311.4	57.3	66.6
13702	317.1	259.9	202.2	756.4	152.8	218.5
13703	78.3	41.3	45.4	314.4	53.4	65.0
13704	208.3	226.3	146.8	848.2	215.7	177.4
13812	725.7	677.0	46.2	905.0	701.3	277.4
Mean	317.2	229.4	156.2	698.3	293.7	180.5

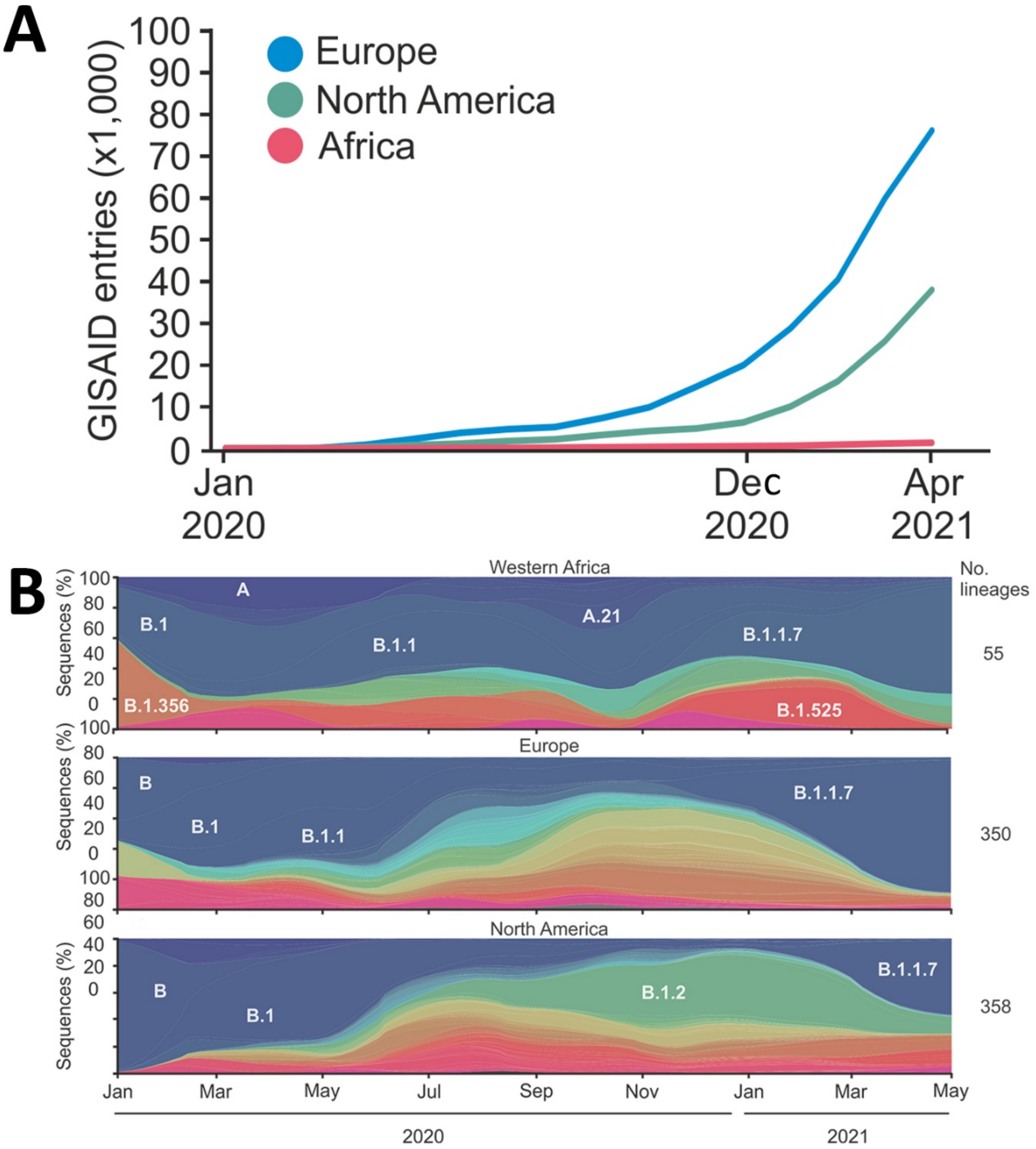

Appendix Figure. Globally available severe acute respiratory syndrome coronavirus 2 (SARS-CoV-2) genomic data. A) SARS-CoV-2 sequence entries from Africa and affluent settings in GISAID on April 24, 2021. B) SARS-CoV-2 genomic epidemiology in West Africa and affluent settings during January 2020–May 2021. Frequency plots were attained from Nextstrain (https://nextstrain.org/ncov/global) on May 25, 2021. The global dataset was filtered for the 3 different regions and their associated countries, including 11 countries for West Africa, 45 countries for Europe, and the United States and Canada for North America. Frequencies are colored by PANGO lineages and normalized to 100% at each time point for 705 sequences from West Africa, 2,400 sequences for Europe, and 1,515 sequences for North America.
